# The Relationship Between Hippocampal Volumes and Delayed Recall Is Modified by APOE ε4 in Mild Cognitive Impairment

**DOI:** 10.3389/fnagi.2019.00036

**Published:** 2019-02-26

**Authors:** Xiwu Wang, Wenjun Zhou, Teng Ye, Xiaodong Lin, Jie Zhang, the Alzheimer’s Disease Neuroimaging Initiative

**Affiliations:** ^1^Department of Psychiatry, Wenzhou Seventh People’s Hospital, Wenzhou, China; ^2^Department of Pathology, Hangzhou Normal University, Hangzhou, China; ^3^Department of Ultrasound, The First Affiliated Hospital of Wenzhou Medical University, Wenzhou, China; ^4^Independent Researcher, Hangzhou, China

**Keywords:** Alzheimer’s disease, amnestic mild cognitive impairment, hippocampus, episodic memory, APOE ε4

## Abstract

**Objective:** To investigate whether APOE ε4 affects the association of verbal memory with neurodegeneration presented by the hippocampal volume/intracranial volume ratio (HpVR).

**Methods:** The study sample included 371 individuals with normal cognition (NC), 725 subjects with amnestic mild cognitive impairment (aMCI), and 251 patients with mild Alzheimer’s disease (AD) from the Alzheimer’s Disease Neuroimaging Initiative (ADNI) who underwent the rey auditory verbal learning test (RAVLT). Multiple linear regression models were conducted to assess the effect of the APOE ε4^∗^HpVR interaction on RAVLT in all subjects and in each diagnostic group adjusting for age, gender and educational attainment, and global cognition.

**Results:** In all subjects, there was no significant APOE ε4 × HpVR interaction for immediate recall or delayed recall (*p* > 0.05). However, in aMCI subjects, there was a significant APOE ε4 × HpVR interaction for delayed recall (*p* = 0.008), but not immediate recall (*p* = 0.15). More specifically, the detrimental effect of APOE ε4 on delayed recall altered by HpVR such that this effect was most evident among subjects with small to moderate HpVR, but this disadvantage was absent or even reversed among subjects with larger HpVR. No significant interaction was observed in the NC or AD group.

**Conclusion:** These findings highlight a potential role of APOE ε4 status in affecting the association of hippocampus size with delayed recall memory in the early stage of AD.

## Introduction

The effect of the apolipoprotein E ε4 (APOE ε4) allele on cognitive abilities is complicated. Previous studies demonstrated the diverse roles of APOE ε4 allele in cognitive abilities dependent on different ages. In young populations, the APOE ε4 allele has a beneficial effect on learning and memory ability. However, the APOE ε4 allele is also associated with the decline of learning and memory ability in old subjects (Han and Bondi, 2008; Caselli et al., 2009; Tuminello and Han, 2011). These interesting findings have been conceptualized as the APOE antagonistic pleiotropy hypothesis (Han and Bondi, 2008; Caselli et al., 2009; Tuminello and Han, 2011).

The hippocampus is critical for episodic memory function and its size is considered as an index of the degree of cognitive decline (Kilpatrick et al., 1997; Eldridge et al., 2000; Ystad et al., 2009). A previous study also showed that the APOE ε4 allele contributes to the reduction of hippocampal volume in patients with mild cognitive impairment (MCI) and Alzheimer’s disease (AD) (Khan et al., 2017). Additionally, APOE ε4 was found to reduce hippocampal volumes and impair episodic memory in a dose-dependent manner (APOE ε4 homozygous > APOE ε4 heterozygous > APOE ε4 non-carriers) (Liu et al., 2016). A recent study revealed that hippocampal volume was positively associated with episodic memory in cognitively normal old individuals with APOE ε4 homozygotes (Lim et al., 2017). However, it is unknown whether the APOE ε4 allele also has antagonistic pleiotropic effects on the association of hippocampal volumes with episodic memory across the AD continuum. Our primary goal was to examine whether the APOE ε4 allele modulates the relationship between hippocampal volumes and verbal memory in subjects with NC, MCI, and mild AD from the Alzheimer’s disease neuroimaging initiative (ADNI) dataset.

## Materials and Methods

### Alzheimer’s Disease Neuroimaging Initiative

Demographic and imaging data used in the preparation of this study were extracted from the ADNI database^1^ in January 2017. The primary aim of the ADNI has been to test whether clinical and neuropsychological assessment, neuroimaging and other biomarkers can be integrated to predict the progression of MCI and early AD. The ADNI was conducted after institutional review board approval at each site. Written informed consent was obtained from all participants or their authorized representatives. Our study sample included subjects who had hippocampal volumetric, verbal memory and APOE 4ε genotype data available from one visit cycle (*n* = 1347). In this study, there were 371 subjects with NC, 725 patients with aMCI, and 251 patients with AD.

### Diagnosis Criteria

Inclusive and exclusive criteria can be found in detail at http://www.adni-info.org. The NC group had a score of at least 24 on the mini-mental state examination (MMSE) and a score of 0 on the Clinical Dementia Rating (CDR) scale (Petersen et al., 2010). The aMCI group had a score of 24 or higher on the MMSE, a CDR score of 0.5, a subjective memory complaint, objective memory impairment as examined by the Logical Memory II subscale of the Wechsler Memory Scale–Revised (Wechsler, 1987), essentially preserved activities of daily living, and were not demented (Petersen et al., 2010). The AD group met the National Institute of Neurological and Communicative Disorders and Stroke and AD and Related Disorders Association criteria for probable AD, having scores from 20 to 26 on the MMSE and a score of 0.5 or 1 on the CDR (Petersen et al., 2010).

### Neuropsychological Outcomes

The MMSE and CDR were used to evaluate global cognitive function and dementia severity, respectively. The rey auditory verbal learning test (RAVLT) (Schmidt, 1996) was applied to measure verbal memory. The immediate recall scores (range, 0–75) and delayed recall scores (range, 0–15) were utilized as our main evaluation indicators.

### Structural Magnetic Resonance Imaging

Structural MRI brain scans were obtained using 1.5T MRI scanners with a standardized protocol, which is described in detail at www.loni.ucla.edu/ADNI. Hippocampal volume measures were performed using FreeSurfer software^2^. In order to correct for subject differences in head size, hippocampal volumes were normalized by individual intracranial volume (formula: hippocampal/intracranial volume × 10^3^). The MRI volumes of brain structures used in this study were extracted from UCSF data in the ADNI dataset^3^.

### APOE Genotyping

The data of APOE genotypes of our study sample were extracted from the ADNI database. Further information can be found at adni.loni.usc.edu. Participants were classified as APOE ε4-negative (-) if they carried no APOE ε4 allele or APOE ε4-positive (+) if they carried at least one APOE ε4 allele.

### Statistical Analysis

Socio-demographics and clinical outcomes were compared between diagnostic groups using analysis of variance (ANOVA) for continuous variables and chi-squared for categorical variables in all subjects and within diagnostic groups. The relationship between HpVR and memory outcomes was analyzed by Pearson correlation analysis in all subjects and within diagnostic groups. HpVR and memory outcomes were compared between APOE ε4 carriers and non-carriers using student’s t test in all subjects and within diagnostic groups. The multivariable linear regression was performed to evaluate the independent and interactive relationship of APOE ε4 status and HpVR on verbal memory performance (RAVLT immediate and delayed recall scores) in all subjects and within diagnostic groups. In model 1, we evaluated the independent effects of APOE ε4 status and HpVR. Then, the HpVR × APOE ε4 status interaction was added to model 2, but was eliminated if not significant (*p* > 0.05). All analyses were adjusted for age, gender, education, MMSE scores and diagnosis (only in the overall sample analysis). The resultant *p*-values for the associations of the APOE ε4^∗^ HpVR interaction with memory performance were corrected for multiple comparisons with false discovery rate (FDR) (Benjamini and Hochberg, 1995). IBM SPSS version 20 was used to perform all statistical analyses. A two-tailed *P* value of less than 0.05 was considered to be statistically significant.

## Results

### Socio-Demographic Data

Table 1 listed the socio-demographic characteristics of the subjects. Several demographic and clinical variables differed significantly across the three diagnostic groups (Table 1).

**Table 1:** Overall sample characteristics by diagnostic groups.

Characteristics	Controls (*n* = 371)	aMCI (*n* = 725)	AD (*n* = 251)	*P* value
Age, y	74.7 (5.6)	72.6 (7.4)^b^	74.7 (8.0)	<0.001
Gender, % male	50.9	58.1	56.2	0.079
APOE ε4, % positive	26.1^a^	50.5^b^	68.9^c^	<0.001
Education, y	16.2 (2.7)	15.9 (2.9)	15.2 (2.9)^c^	<0.001
MMSE, score	29.1 (1.1)^a^	27.6 (1.8)^b^	23.1 (2)^c^	<0.001
RAVLT immediate recall	44.5 (9.8)^a^	34.8 (10.8)^b^	23.3 (7.1)^c^	<0.001
RAVLT delayed recall	5.8 (2.3)^a^	4.3 (2.5)^b^	2.0 (1.6)^c^	<0.001
Hippocampal volume, mm^3^	7355 (907)^a^	6787 (1137)^b^	5789 (1032)^c^	<0.001
HpVR	4.9 (0.6)^a^	4.4 (0.8)^b^	3.8 (0.7)^c^	<0.001

*MMSE, mini-mental state examination; RAVLT, rey auditory verbal learning test; aMCI, amnestic mild cognitive impairment; AD, Alzheimer’s disease; HpVR, hippocampal volume ratio (hippocampal/intracranial volume × 1000). Data are summarized as mean (SD). Alphabetic “a,” “b,” and “c” superscripts show that the pairwise groups have statistical significance using the Tukey HSD*.

### Association of HpVR With RAVLT

To examine the relationships between HpVR and RAVLT immediate and delayed recall scores, Pearson correlation tests were performed in all subjects and within three diagnostic groups (Table 2). As expected, positive correlations between HpVR and RAVLT immediate (*r* = 0.485, *p* < 0.001) and delayed (*r* = 0.413, *p* < 0.001) recall scores were found in all subjects. In diagnosis-stratified analyses, a positive correlation between HpVR and immediate recall (*r* = 0.117, *p* = 0.024), but not delayed recall (*r* = 0.044, *p* = 0.4), was observed in controls. In agreement with findings in the whole sample, HpVR was positively correlated with immediate recall (*r* = 0.414, *p* < 0.001), and delayed recall (*r* = 0.341, *p* < 0.001) scores in subjects with MCI. Among subjects with AD dementia, HpVR was correlated with immediate recall (*r* = 0.152, *p* = 0.016), and delayed recall (*r* = 0.229, *p* < 0.001) scores.

**Table 2:** Association between RAVLT and HpVR in three diagnostic groups.

	HpVR							
	All subjects		Controls		aMCI		AD	
	*r*	*p*	*r*	*p*	*r*	*p*	*r*	*p*
Immediate recall	0.485	<0.001	0.117	0.024	0.414	<0.001	0.152	0.016
Delayed recall	0.413	<0.001	0.044	0.4	0.341	<0.001	0.229	<0.001

*RAVLT, rey auditory verbal learning test; aMCI, amnestic mild cognitive impairment; AD, Alzheimer’s disease; HpVR, hippocampal volume ratio (hippocampal/intracranial volume × 1000)*.

### Effect of APOE ε4 Genotypes on HpVR and RAVLT

In the overall sample, APOE ε4 carriers had lower RAVLT immediate and delayed recall scores and smaller hippocampal volumes compared to non-carriers (Table 3). In diagnosis-stratified analyses, APOE ε4 carriers had lower RAVLT immediate and delayed recall scores in the MCI group, but not the NC or AD group. Further, compared with non-carriers, APOE ε4 carriers had smaller hippocampal volumes in the MCI and AD groups, but not the NC group.

**Table 3:** RAVLT and HpVR by diagnostic groups and APOE ε4 status.

	APOE ε4-	APOE ε4+	*p* value
**All subjects**			
RAVLT immediate recall	38.0 (12.1)	32.3 (11.7)	<0.001
RAVLT delayed recall	4.8 (2.6)	3.7 (2.6)	<0.001
HpVR	4.6 (0.8)	4.3 (0.8)	<0.001
**Controls**			
RAVLT immediate recall	44.4 (9.4)	44.8 (10.6)	0.759
RAVLT delayed recall	5.9 (2.2)	5.7 (2.5)	0.47
HpVR	4.9 (0.6)	4.9 (0.7)	0.656
**aMCI**			
RAVLT immediate recall	36.3 (11.4)	33.3 (10.1)	<0.001
RAVLT delayed recall	4.6 (2.5)	4.0 (2.6)	0.001
HpVR	4.53 (0.8)	4.36 (0.8)	0.005
**AD**			
RAVLT immediate recall	23.4 (7.0)	23.3 (7.1)	0.92
RAVLT delayed recall	2.2 (1.8)	2.0 (1.5)	0.218
HpVR	3.9 (0.8)	3.7 (0.6)	0.031

*RAVLT, rey auditory verbal learning test; aMCI, amnestic mild cognitive impairment; AD, Alzheimer’s disease; HpVR, hippocampal volume ratio (hippocampal/intracranial volume × 1000)*.

### Linear Regression Results

**Figure 1:**
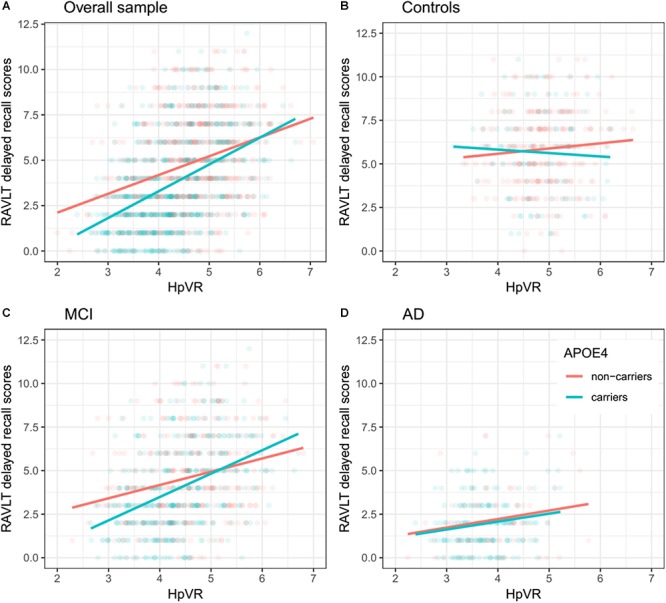
Association between HpVR and rey auditory verbal learning test (RAVLT) delayed recall scores in APOE ε4 carriers and non-carriers. RAVLT delayed recall scores as a function of HpVR (hippocampal/intracranial volume × 10^3^) and APOE ε4 status in the **(A)** overall sample, **(B)** controls, **(C)** amnestic mild cognitive impairment (aMCI), and **(D)** Alzheimer disease (AD) dementia.

In all subjects, there was no significant HpVR by APOE ε4 status interaction for immediate recall (*p* = 0.26; Table 4) or delayed recall (*p* = 0.091; Table 4 and Figure 1A). In diagnosis-stratified analyses, the HpVR by APOE ε4 status interaction was significant for delayed recall in an aMCI group (*p* = 0.008), but not immediate recall (*p* = 0.15). More specifically, the association between HpVR and delayed recall was stronger in APOE ε4 carriers compared to non-carriers. Figure 1C shows that APOE ε4 carriers outperform non-carriers on delayed recall memory among subjects with large HpVR (right side of the x-axis) but this advantage gradually disappears and reverses to confer memory deficits among subjects with moderate to small HpVR (left side of the x-axis). However, the association of the APOE4^∗^HpVR interaction with delayed recall scores in the MCI group did not survive FDR correction (*p* = 0.096). The HpVR by APOE ε4 status interaction for immediate recall and delayed recall was not significant in the control or in the AD dementia group (all *p* > 0.05, Table 4 and Figure 1B,D). Among controls, no significant difference in immediate (*p* = 0.8) or delayed recall (*p* = 0.3) was found between APOE ε4 carriers and non-carriers, and HpVR was not associated with immediate recall (*p* = 0.7) or delayed recall (*p* = 0.6) (Table 4). In AD dementia, no significant difference in immediate (*p* = 0.7) and delayed recall (*p* = 0.34) was found between APOE ε4 carriers and non-carriers. In AD dementia, larger HpVR was associated with better delayed recall (*p* = 0.014), but not with immediate recall (*p* = 0.4).

**Table 4:** Results of multivariable linear regression analyses modeling the independent and interactive effects of APOE ε4 status and HpVR on verbal memory performance.

Sample/outcome	Multivariable linear regression models					
	Model 1: No interactions in model					
	APOE ε4 status (- vs. +)		HpVR		Model 2: Interaction included in model, APOE ε4 status^∗^HpVR	
	B (SE)	*P* value	B (SE)	*P* value	B (SE)	*P* value
**Overall sample**					
Immediate recall	-1.2 (0.5)	0.016	2.2 (0.37)	<0.001	0.7 (0.6)	0.26
Delayed recall	-0.3 (0.1)	0.017	0.6 (0.1)	<0.001	0.3 (0.2)	0.091
**Controls**						
Immediate recall	-0.3 (1.1)	0.8	-0.3 (0.9)	0.7	0.4 (1.7)	0.8
Delayed recall	-0.3 (0.3)	0.3	-0.1 (0.2)	0.6	-0.6 (0.4)	0.2
**aMCI**						
Immediate recall	-1.8 (0.7)	0.006	3.1 (0.5)	<0.001	1.2 (0.8)	0.15
Delayed recall	-0.3 (0.2)	0.088	0.8 (0.1)	<0.001	0.6 (0.2)	0.008
**AD dementia**						
Immediate recall	0.3 (0.9)	0.7	0.6 (0.8)	0.4	-0.4 (1.3)	0.77
Delayed recall	-0.2 (0.2)	0.34	0.4 (0.2)	0.014	-0.1 (0.3)	0.66

*AD, Alzheimer disease; aMCI, amnestic mild cognitive impairment; B, unstandardized regression coefficient; HpVR, hippocampal volume ratio (hippocampal/intracranial volume × 1000). All analyses were adjusted for age, sex, education, MMSE scores, and diagnostic group (overall sample only)*.

**Figure 2:**
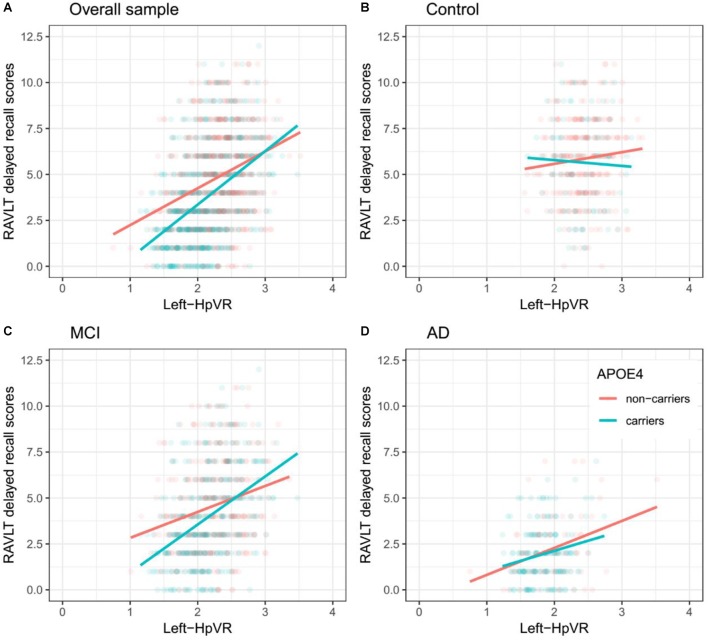
Association between left-HpVR and RAVLT delayed recall scores in APOE ε4 carriers and non-carriers. RAVLT delayed recall scores as a function of Left-HpVR (hippocampal/intracranial volume × 10^3^) and APOE ε4 status in the **(A)** overall sample, **(B)** controls, **(C)** aMCI, and **(D)** AD dementia.

**Figure 3:**
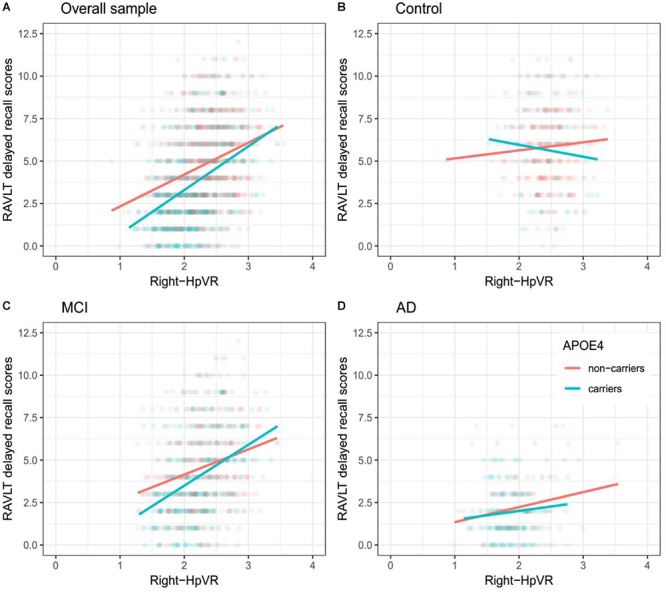
Association between right-HpVR and RAVLT delayed recall scores in APOE ε4 carriers and non-carriers. RAVLT delayed recall scores as a function of Right-HpVR (hippocampal/intracranial volume × 10^3^) and APOE ε4 status in the **(A)** overall sample, **(B)** controls, **(C)** aMCI, and **(D)** AD dementia.

In addition, we also examined the effect of the left-HpVR/Right-HpVR^∗^APOE ε4 interaction on RAVLT immediate and delayed recall scores (see Figure 2, 3 and Tables 5, 6). Similarly, a significant left-HpVR^∗^APOE ε4 interaction for delayed recall among MCI subjects was observed (*p* = 0.004), while the association did not survive FDR correction (*p* = 0.096). Further, there was a significant right-HpVR^∗^APOE ε4 interaction for delayed recall among MCI subjects (*p* = 0.034), but the association did not survive FDR correction (*p* = 0.27).

**Table 5:** Results of multivariable linear regression analyses modeling the independent and interactive effects of APOE ε4 status and left-HpVR on verbal memory performance.

Sample/outcome	Multivariable linear regression models					
	Model 1: No interactions in model					
	APOE ε4 status (- vs. +)		Left-HpVR		Model 2: Interaction included in model, APOE ε4 status^∗^Left- HpVR	
	B (SE)	*P* value	B (SE)	*P* value	B (SE)	*P* value
**Overall sample**					
Immediate recall	-1.2 (0.5)	0.16	4.3 (0.7)	<0.001	1.6 (1.2)	0.17
Delayed recall	-0.3 (0.13)	0.15	1.2 (0.18)	<0.001	0.6 (0.3)	0.054
**Controls**						
Immediate recall	-0.2 (1.1)	0.8	-0.2 (1.6)	0.883	0.02 (3.3)	0.996
Delayed recall	-0.3 (0.3)	0.3	-0.1 (0.4)	0.8	-1 (0.8)	0.2
**aMCI**						
Immediate recall	-1.9 (0.7)	0.006	6 (0.9)	<0.001	2.6 (1.6)	0.1
Delayed recall	-0.3 (0.18)	0.072	1.5 (0.2)	<0.001	1.2 (0.4)	0.004
**AD dementia**						
Immediate recall	0.4 (0.9)	0.7	1.5 (1.5)	0.3	0.6 (2.6)	0.8
Delayed recall	-0.16 (0.2)	0.47	1.2 (0.3)	0.001	-0.3 (0.6)	0.6

*AD, Alzheimer disease; aMCI, amnestic mild cognitive impairment; B, unstandardized regression coefficient; HpVR, hippocampal volume ratio (hippocampal/intracranial volume × 1000). All analyses were adjusted for age, sex, education, MMSE scores, and diagnostic group (overall sample only)*.

**Table 6:** Results of multivariable linear regression analyses modeling the independent and interactive effects of APOE ε4 status and Right-HpVR on verbal memory performance.

Sample/outcome	Multivariable linear regression models				
	Model 1: No interactions in model			
	APOE ε4 status (- vs. +)		Right-HpVR		Model 2: Interaction included in model, APOE ε4 status^∗^Right-HpVR	
	B (SE)	*P* value	B (SE)	*P* value	B (SE)	*P* value
**Overall sample**					
Immediate recall	-1.2 (0.5)	0.015	3.8 (0.7)	<0.001	1 (1.1)	0.37
Delayed recall	-0.3 (0.1)	0.11	0.9 (0.17)	<0.001	0.3 (0.3)	0.25
**Controls**						
Immediate recall	-0.2 (1.1)	0.8	-0.2 (1.6)	0.9	1 (3)	0.7
Delayed recall	-0.3 (0.3)	0.3	-0.4 (0.4)	0.37	-1.3 (0.8)	0.09
**aMCI**						
Immediate recall	-1.8 (0.7)	0.007	5.6 (0.9)	<0.001	2 (1.6)	0.2
Delayed recall	-0.3 (0.18)	0.088	1.4 (0.2)	<0.001	0.9 (0.4)	0.034
**AD dementia**						
Immediate recall	0.2 (0.9)	0.8	0.5 (1.3)	0.7	-2 (2.4)	0.4
Delayed recall	-0.3 (0.2)	0.2	0.4 (0.3)	0.2	-0.2 (0.6)	0.7

*AD, Alzheimer disease; aMCI, amnestic mild cognitive impairment; B, unstandardized regression coefficient; HpVR, hippocampal volume ratio (hippocampal/intracranial volume × 1000). All analyses were adjusted for age, sex, education, MMSE scores, and diagnostic group (overall sample only)*.

## Discussion

In the present study, we examined the effect of the APOE ε4 allele on the interplay between episodic memory and hippocampal volume in the three diagnostic groups (controls, aMCI, mild AD). Our study found an interesting fact that, depending on different hippocampal volumes, the APOE ε4 allele asserts different effects on delayed recall. Our data suggested that the deleterious effect of APOE ε4 on delayed recall altered by HpVR such that this effect was most evident among aMCI patients with small to moderate HpVR, but this disadvantage was absent or even reversed among aMCI patients with larger HpVR, indicating the dual effects of APOE ε4 on the association of delayed recall memory and hippocampal volumes.

Antagonistic pleiotropy (Williams, 1957) posits that certain genes or alleles may affect fitness (for instance, survival, and reproduction) differentially at different ages. Recently, the antagonistic pleiotropy hypothesis of APOE has been proposed by some researchers based on findings that the impact of the APOE ε4 allele on cognitive functioning, episodic memory in particular, may be beneficial at younger ages, while it appears detrimental in later life (Han and Bondi, 2008; Caselli et al., 2009; Tuminello and Han, 2011; Jochemsen et al., 2012). However, concomitant underlying mechanisms of these changes remain unclear. Increasing evidence suggested that APOE ε4 carriers may primarily recruit greater compensatory resources to maintain equivalent or superior levels of memory performance as APOE ε4 non-carriers. Nevertheless, once AD pathological burdens sufficiently accumulate, APOEε4 carriers’ compensatory recruitment will fail to sustain memory performance and memory decline ensues (Han and Bondi, 2008). For instance, one study (Bookheimer et al., 2000) using fMRI techniques suggested that APOE ε4 carriers, in non-demented older people, showed greater activation in the hippocampus and other brain regions during the encoding portion of a memory task, providing evidence for APOEε4 carriers’ compensatory recruitment. In other words, APOE ε4 carriers may more greatly activate memory-related brain regions to sustain the same or better level of cognitive performance as APOE ε4 non-carriers. Dickerson et al. (2005) also reported findings supportive of APOEε4 carriers’ compensatory recruitment in three diagnostic groups, including cognitively normal older people and patients with MCI or AD. They found that APOEε4 carriers, irrespective of diagnosis, showed increased hippocampal activity during the encoding portion of a face-name task. Similarly, a more recent study suggested that APOEε4 carriers recruited additional neural resources to successfully complete a challenging working memory task (Scheller et al., 2017). However, once the compensatory recruitment of neural resources fails, memory may begin to decline (Han and Bondi, 2008; Tuminello and Han, 2011). Most cross-sectional studies have supported the idea that APOE ε4 carriers in older people performed more poorly than non-carriers in episodic memory tasks (Honea et al., 2009; Adamson et al., 2010; Kukolja et al., 2010), while not universally the case (Welsh-Bohmer et al., 2009). Furthermore, one important study by Caselli et al. (2009) longitudinally followed individuals ranging from 21 to 97 years of age. They found that memory decline in APOEε4 carriers started before 60 years of age and showed a steeper rate of memory decline compared to non-carriers, indicating that the detrimental effects of APOE ε4 on episodic memory may become evident around the age of 60 years. Similarly, based on our findings (Figure 1C), APOE ε4 carriers in the aMCI group, despite similar hippocampal volumes, outperformed non-carriers on episodic memory among subjects with large HpVR (HpVR > approximately 5.2, referring to the compensatory recruitment state). Once AD pathological burdens sufficiently accrue (such as hippocampal atrophy), this APOE ε4 advantage was not evident and gradually reversed to confer memory deficits among subjects with moderate to small HpVR (HpVR < approximately 5.2, referring to the failure of compensatory recruitment). Our data indicated that APOE ε4 carriers as an at-risk population for AD may benefit from drug or non-drug interventions that are tailored to the levels of hippocampal atrophy. After acquisition of APOE ε4 genotype and hippocampal volume data, APOE ε4 carriers could be further targeted by some interventions to maintain memory performance at this paramount turning point.

Our findings have several important implications. The dual effects of APOE ε4 on the association of delayed recall memory with hippocampal volumes are clinically critical because delayed recall memory is used to diagnose aMCI and AD dementia (Albert et al., 2011; McKhann et al., 2011; Dubois et al., 2014) and norms of episodic memory tests are currently not APOE ε4-adjusted. Therefore, this may lead to some misdiagnoses if we do not take APOE ε4 status into consideration. For instance, due to our findings (Figure 1C), a true aMCI diagnosis may be more likely to be delayed in APOE ε4 carriers than non-carriers in individuals with HpVR > approximately 5.2, because beneficial effects of APOE ε4 on episodic memory in these subjects may mask underlying AD neuropathology, particularly in the earlier stages of disease.

There are several potential limitations in the present study. First, the cross-sectional design prevents us from determining temporality in the association between episodic memory and HpVR. In this cross-section analysis, we also could not measure rates of memory decline in APOE ε4 carriers vs. non-carriers. Longitudinal studies are needed to more accurately test the hypothesis that APOE ε4 status may modulate the relationship between hippocampus volumes and episodic memory. Second, the APOE antagonistic pleiotropy hypothesis proposes that the APOE ε4 allele is linked to better cognitive functioning in young adulthood and then it reverses to confer cognitive deficits in older age. However, in the current study, the lack of younger people limits our test of the APOE antagonistic pleiotropy hypothesis. Third, the HpVR by APOE ε4 interaction for memory testing was not significant in the control or in the AD dementia group. One potential explanation may be due to the fact that the variability of memory scores and hippocampal volumes may be low in controls, and memory scores and hippocampal volumes may have reached a plateau in AD patients. Finally, after FDR correction, a marginally significant two-way APOE ε4^∗^HpVR interaction for delayed recall in MCI subjects was observed (*p* = 0.096), and thus this finding requires replication.

In summary, our study highlights the potential role of APOE ε4 in affecting the relationship between hippocampus volumes and delayed recall memory in MCI patients.

## Data Availability

Publicly available datasets were analyzed in this study. This data can be found here: http://adni.loni.usc.edu/.

## Ethics Statement

All subjects gave signed, informed consent to participate in the study. Clinical research described in the manuscript was carried out in accordance with Declaration of Helsinki promulgated by the National Institute of Health.

## Author Contributions

JZ and XL conceived and designed the study. XW, WZ, and TY performed the research, analyzed the data, and wrote the manuscript.

## Conflict of Interest Statement

The authors declare that the research was conducted in the absence of any commercial or financial relationships that could be construed as a potential conflict of interest.

## Footnotes

1 http://adni.loni.usc.edu

2 http://surfer.nmr.mgh.harvard.edu

3 https://ida.loni.usc.edu/pages/access/studyData.jsp
